# Design and Regulation of Novel MnFe_2_O_4_@C Nanowires as High Performance Electrode for Supercapacitor

**DOI:** 10.3390/nano9050777

**Published:** 2019-05-21

**Authors:** Lei Geng, Fengfeng Yan, Chenhao Dong, Cuihua An

**Affiliations:** 1 Tianjin Key Laboratory of Optoelectronic Detection Technology and Systems, School of Electronics and Information Engineering, Tianjin Polytechnic University, No. 399 Binshui West Street Xiqing District, Tianjin 300387, China; tjpuyff@163.com; 2 Tianjin Key Laboratory of Advanced Functional Porous Materials, Institute for New Energy Materials & Low-Carbon Technologies, School of Materials Science and Engineering, Tianjin University of Technology, No. 391 Binshui West Street Xiqing District, Tianjin 300384, China; dong15620373251@163.com (C.D.); ancuihua@tjut.edu.cn (C.A.)

**Keywords:** in-situ carbon coating, MnFe_2_O_4_ nanowire, supercapacitor, long-term stability

## Abstract

Bimetallic oxides have been considered as potential candidates for supercapacitors due to their relatively high electric conductivity, abundant redox reactions and cheapness. However, nanoparticle aggregation and huge volume variation during charging-discharging procedures make it hard for them to be applied widely. In this work, one-dimensional (1D) MnFe_2_O_4_@C nanowires were in-situ synthesized via a simply modified micro-emulsion technique, followed by thermal treatment. The novel 1D and core-shell architecture, and in-situ carbon coating promote its electric conductivity and porous feature. Due to these advantages, the MnFe_2_O_4_@C electrode exhibits a high specific capacitance of 824 F·g^−1^ at 0.1 A·g^−1^ and remains 476 F·g^−1^ at 5 A·g^−1^. After 10,000 cycles, the capacitance retention of the MnFe_2_O_4_@C electrode is up to 93.9%, suggesting its excellent long-term cycling stability. After assembling with activated carbon (AC) to form a MnFe_2_O_4_@C//AC device, the energy density of this MnFe_2_O_4_@C//AC device is 27 W·h·kg^−1^ at a power density of 290 W·kg^−1^, and remains at a 10 W·h·kg^−1^ energy density at a high power density of 9300 W·kg^−1^.

## 1. Introduction

Electrochemical supercapacitors have been in the research spotlight owing to their intriguing characteristics, such as high power density, wide temperature ranges, long cycling stability and safety [1,2,3,4]. Despitethe aforementioned prominent advantages, the large-scale utilization for supercapacitors is restricted by their low energy density [5,6,7]. Due to the close relationship between energy density with the specific capacitance and working voltage, designing a composite electrode possessing high specific capacitance and fabricating asymmetric supercapacitors (ASC) with broad working voltage is deemed to be the most useful way [8,9,10,11,12]. 

One of the effective strategies to improve electrochemical performances of the ASC is to synthesize peculiar electrode materials. Among the various electrode materials, bimetallic oxide materials, especially MnFe_2_O_4_, have aroused attention in the past few decades on account of their cheapness, environmental amity, and plentiful oxidation valences [13,14,15,16]. Thus, MnFe_2_O_4_ materials with plentiful morphologies and structures have been prepared and used as supercapacitor electrodes. But the electrochemical performances of these MnFe_2_O_4_ materials were barely satisfactory due to easy aggregation and volume expansion during the charging-discharging process, and low electrical conductivity. Up to now, many efforts have been devoted to fabricating carbon-metal oxide composites, which can improve the electrical conductivities of the bimetallic oxides and enhance the rate properties of electrodes at the same time. MnFe_2_O_4_@C (such as graphene, rGO and carbon black, etc.) nanocomposites have been widely studied [17,18,19,20]. Although the electrochemical performances of MnFe_2_O_4_@C composites have been effectively enhanced through tuning the morphology and porous structure, the preparation procedures usually experience some rough or tedious steps, with high energy consumption and toxic or unfriendly effects to the environment. How to prepare MnFe_2_O_4_@C composites with uniform carbon coating via a facile, green and cheap route is a matter of urgency. 

Herein, MnFe_2_O_4_@C nanowires have been in-situ synthesized by a micro-emulsion approach. The particular architecture, nanowire in whole and core-shell in part, are poriferous but extremely sturdy, promoting electron and ion transportation for redox reactions. The uniform carbon coating not only improves the electric conductivity, but also protects the core-shell nanoparticles from aggregation during repeated charging-discharging processes. Profiting from these superiorities, the MnFe_2_O_4_@C composites deliver a specific capacitance of 824 F·g^−1^ at 0.1 A·g^−1^ and maintain 476 F·g^−1^ at 5 A·g^−1^, indicating 57.8% capacitance retention. Moreover, the MnFe_2_O_4_@C electrode exhibits a prominent cycling stability of 93.9% after 10,000 cycles at 1 A·g^−1^.

## 2. Materials and Methods

**Scheme 1:**
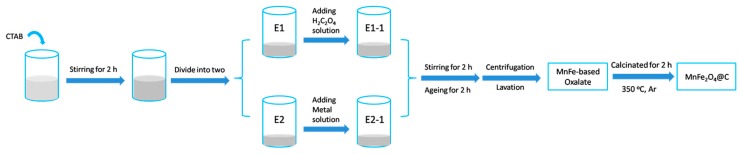
Schematic illustration of the prepared MnFe_2_O_4_@C composite.

The micro-emulsion approach was modified in this work to prepare MnFe_2_O_4_@C composites (Scheme 1) with a modified procedure [21,22,23]. More specifically, 0.5 mmol cetyltrimethyl ammonium bromide (CTAB) was put into the mixed solution of pentanol and cyclohexane (volume ratio of 1:10), and stirred for 2 h to generate a homogeneous micro-emulsion. The above micro-emulsion should be in duplicate, named as E1 and E2. Subsequently, 2 M H_2_C_2_O_4_ (5 mL) solution was continuously dropped to the E1, designated as E1-1. And 0.1 M MnSO_4_ and 0.2 M (NH_4_)_2_Fe(SO_4_)_2_ solution was added into the E2 to form E2-1. Moreover, The E1-1 solution was mixed with E2-1 under persistent stirring for 2 h. Then the mixed micro-emulsion was aged for another 2 h. The sediment (MnFe-based oxalate) was collected by centrifugation, lavaged with pure water and ethanol a few times and dried in an oven overnight. The dried sediment was calcinated at 350 °C for 2 h under Ar atmosphere to receive the endproduct. 

The thermodynamic property of the sediment was measured by the TG (SETAR-AM, SENSYS EVO, TA Instruments, Newcastle, DE, America). The measurement conditions of the TG were as follows: the heating rate, the test temperature range and the atmosphere are 10 °C·min^−1^, 20 to 800 °C and Ar with 99.99% purity. The chemical constitution, valances and morphology were characterized by X-ray diffraction (XRD, Rigaku D/Max-2500, Cu Kα radiation, Tokyo, Japan), X-ray photoelectron spectroscopy (XPS, Escalab250Xi, Thermo Scienticfic, England), Raman spectroscopy (532 nm laser excitation wavelength, Horiba Evolution, Horiba JobinYvon S.A.S., Paris, France) and field-emission scanning electron microscopy (FESEM, FEI Verios 460 L, 5.00 kV accelerating voltage, Eindhoven, The Netherlands), and field-emission transition electron microscope (FETEM, FEI, Technai G2 Spirit TWIN, 120 kV accelerating voltage, Eindhoven, The Netherlands). 

The detailed process for the fabrication of the working electrode was as follows: 1) 24 mg active materials MnFe_2_O_4_@C, 3 mg acetylene black (AB) and 3 mg polyvinylidene fluoride (PVDF) were mixed in a mortar. Then, 250 μL N-methyl-2-pyrrolidinone (NMP) and 600 μL ethanol were added into the above mortar. The homogenous slurry was formed after manual-milling with the pestle for 30 min. 2) 10 μL above homogenous slurry was uniformly dropwise added into carbon foam (1 cm × 1 cm) with a dropper. 3) The above carbon foam was dried in an oven at 80 °C for 12 h. In our working, the mass of the active materials MnFe_2_O_4_@C is about 1.5 mg. The electrochemical tests were implemented in a three-electrode system. The reference, counter and working electrodes were saturated calomel electrode (SCE), Pt and MnFe_2_O_4_@C electrodes, respectively. And 2 M KOH aqueous solution was the electrolyte. For the two-electrode measurement, activated carbon (AC) was used as the cathode. AC was fabricated into an electrode according to the above fabrication procedure of the MnFe_2_O_4_@C electrode without adding AB. The electrolyte was 2 M KOH aqueous solution and the separator membrane was glass fiber. The MnFe_2_O_4_@C electrode and AC electrode were applied to assemble two-electrode cells in coin-cells. Cyclic voltammetry (CV) and galvanostatic charge-discharge (GCD) of the MnFe_2_O_4_@C electrode and the MnFe_2_O_4_@C//AC device were conducted on the electrochemical workstation (CHI760E, Shanghai Chenhua Instrument co., LTD, China) and LAND (2001A, Wuhan LAND Electronic co., LTD, China), respectively.

The specific capacitance of electrode or device are calculated by integrating the CV curves according to the following Equation (1):

(1) $$C=\frac{1}{\mathrm{mv}\left(V_{f}-V_{i}\right)}\int_{V_{i}}^{V_{f}}I\left(V\right)\mathrm{dV}$$

where C (F·g^−1^) is the specific capacitance, m (g) is the mass of the active materials (MnFe_2_O_4_@C) of the electrode or the total mass of the device, v (V·s^−1^) is the scan rate, V_i_ and V_f_ (V) are the initial and final potentials in the CV curves, respectively, and I (A) is the corresponding current.

The specific capacitance of the electrode is calculated from GCD plots according to the following Equation (2):

(2) $$C=\frac{I△t}{m△V}$$

where C (F·g^−1^) is the specific capacitance, △t (s) is the discharging time, m (g) is the mass of the electrode or the total mass of the device, and △V (V) is the working voltage window.

## 3. Results and Discussion

**Figure 1:**
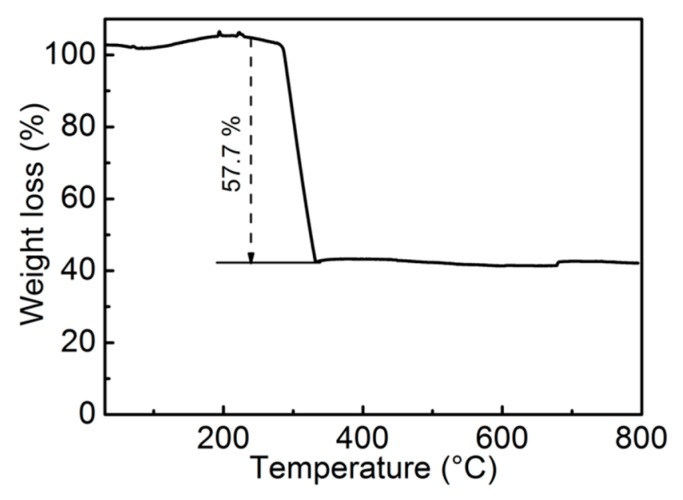
TG curve of the precursor MnFe-based oxalate.

The thermodynamic property of the precursor MnFe-based oxalate is depicted in Figure 1. Obviously, there was a sharp argavic peak with the increase of the heating temperature. It occurred from 275 °C to 325 °C, corresponding to the decomposition of the MnFe-based oxalate. On account of the TG results, the calcinated condition was maintained at 350 °C for 2 h to ensure its complete decomposition. 

**Figure 2:**
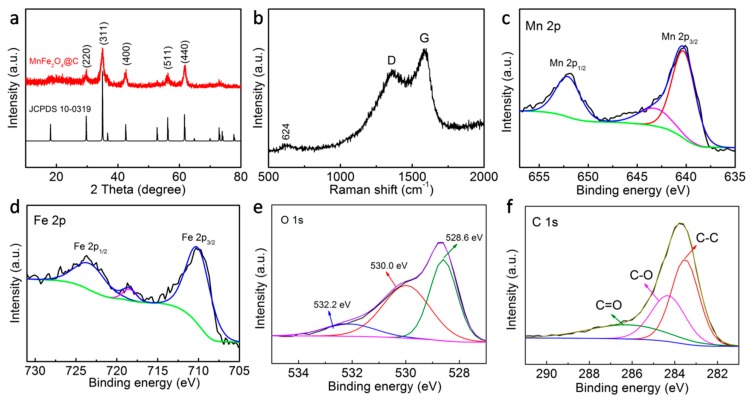
XRD pattern (**a**), Raman spectrum (**b**), XPS spectra for Mn 2p (**c**), Fe 2p (**d**), O 1s (**e**) and C 1s (**f**) of the MnFe_2_O_4_@C composite.

The chemical constitution and valances of the MnFe_2_O_4_@C products are displayed in Figure 2. All the diffraction peaks, except for the broad peak at around 20°, belong to the cubic MnFe_2_O_4_ (JCPDS No. 10-0319) (Figure 2a). The broad peak confirms the presence of the amorphous carbon [24,25]. Moreover, the degree of graphitization for carbon in MnFe_2_O_4_@C composites has been further verified by the Raman spectrum (Figure 2b). The obvious peak at 624 cm^−1^ can be assigned to the characteristic peak of MnFe_2_O_4_, further confirming its successful preparation [26,27]. The featured peaks of D- and G-bands appear at 1380 and 1590 cm^−1^, respectively, standing for amorphous and graphitized carbon. And the I_D_/I_G_ ratio after calculation is about 0.81, which is far away from the fully graphitic level (0.09), suggesting its disorder characteristic and high electric conductivity [28,29,30]. The Raman result is accordant with the XRD analysis. The XPS spectrum of Mn 2p in Figure 2c presents two main peaks at 652.3 and 640.4 eV, ascribing to Mn 2p_1/2_ and Mn 2p_3/2_, respectively. The energy differences of these two peaks is about 11.9 eV, which is on the verge of the numerical value for Mn_3_O_4_, indicating the coexistence of Mn^2+^ and Mn^3+^ ions [28,29]. The remaining peak at 643.6 eV is the Mn 2p satellite peak. Similarly, the XPS spectrum of Fe 2p can be fitted into two peaks at 723.8 and 710.4 eV, rooting in Fe^2+^ and Fe^3+^ ions, respectively (Figure 2d). The satellite peak of the Fe 2p appears at 718.6 eV [28,29]. There are three obvious fitted peaks at 528.6, 530.0 and 532.2 eV in Figure 2e, which correspond to the metallic oxides and sorbed H_2_O [30,31]. Similarly, the C 1s spectra for the MnFe_2_O_4_@C composites can be fitted to three peaks, corresponding to C-C, C-O and C=O [30,31].

**Figure 3:**
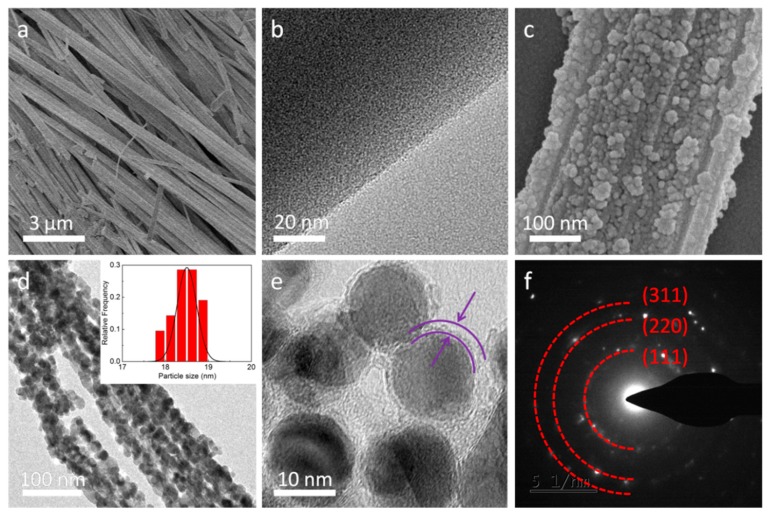
SEM (**a**) and TEM (**b**) image of the MnFe-based oxalate, SEM image (**c**), TEM images (**d**,**e**) and SAED pattern (**f**) of the MnFe_2_O_4_@C composite.

Figure 3 reveals the SEM and TEM images of the MnFe-based oxalate and MnFe_2_O_4_@C composite. As designed, the structure of the MnFe-based oxalate obtained via a modified micro-emulsion technique is 1D nanowire (Figure 3a). And the diameter and length of the MnFe-based oxalate nanowire are about 200 nm and 10 μm, respectively. In addition, the surfaces of these nanowires are smooth (Figure 3b). After calcination, the MnFe_2_O_4_@C composites preserve the whole structure of nanowire, while they present a rough surface compared with the metal oxalate (Figure 3c). The MnFe_2_O_4_@C composites are composed of innumerable nanoparticles, which is demonstrated by the TEM results (Figure 3d). The size distribution of the MnFe_2_O_4_@C composites is displayed in the inset of Figure 3c. In the high-resolution TEM image (Figure 3e), the core-shell structures were constructed with 15 nm MnFe_2_O_4_ core and 3 nm carbon coating. The selected area electron diffraction (SAED) pattern of MnFe_2_O_4_@C composites has been added in Figure 3f and presents three diffraction rings, which correspond to (111), (220) and (311) planes, and illustrates the polycrystalline structure. This original nanostructure is conducive to transferring electronics fast, infiltrating into the active materials and tolerating the volume change during the charging-discharging processes.

**Figure 4:**
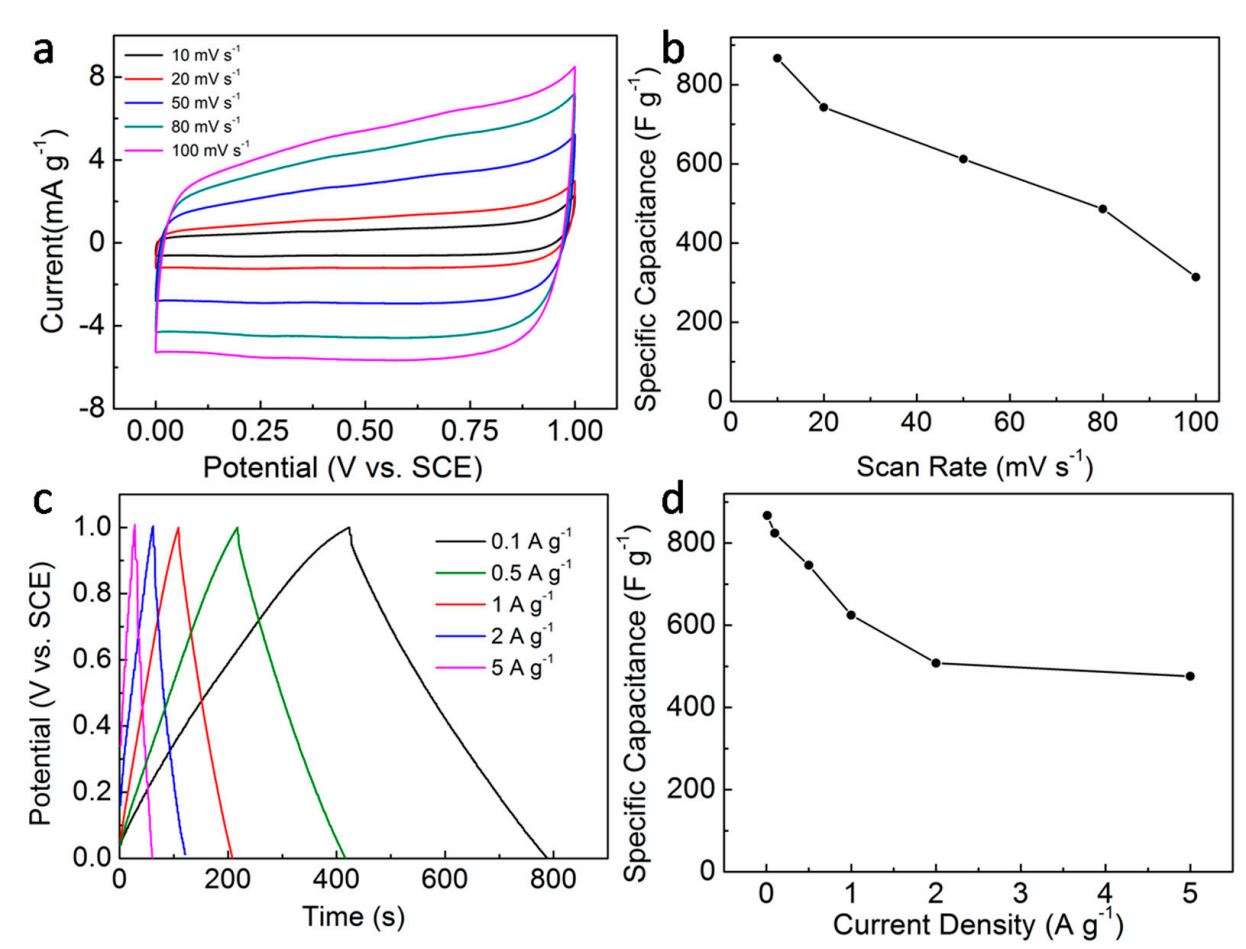
CV curves (**a**), the corresponding specific capacitances at various scan rates (**b**), GCD plots (**c**), the corresponding specific capacitances at various current densities (**d**) of the MnFe_2_O_4_@C electrode.

**Figure 5:**
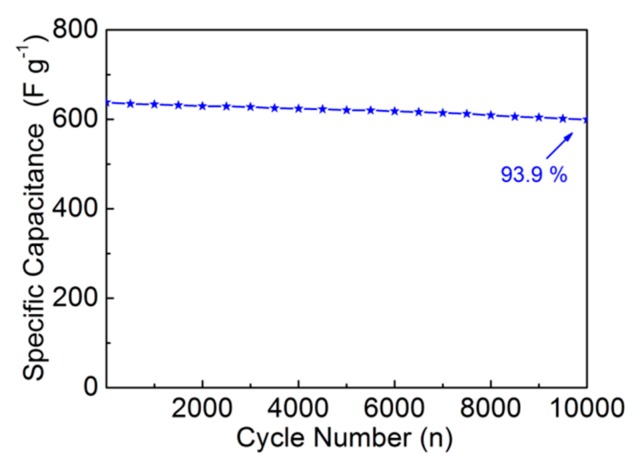
Long-term cycling performance of the MnFe_2_O_4_@C electrode.

CV curves of the MnFe_2_O_4_@C electrode at the scan rates from 10 to 100 mV·s^−1^ are displayed in Figure 4a. The whole shape of the various CV curves is near-rectangle, which is close to the ideal pseudocapacitor with rectangle CV curve. The CV curves are nearly symmetric, demonstrating its high reversibility of the electrochemical reaction. The near-rectangle shape has not been deformed at a high scan rate, suggesting its excellent kinetic performances. Moreover, by increasing the scan rates, the response current density enhances significantly, which could be because the ions in electrolytes infiltrate into the active materials shorter at a high scan rate. Note that the contribution of the carbon foam to the specific capacitance can be ignored [32]. The calculated specific capacitances with scan rates are 867, 743, 612, 486 and 314 F·g^−1^ (Figure 4b), which is consistent with the previous literatures [33,34]. The decrease in capacitance of the MnFe_2_O_4_@C electrode with the increase of scan rate may be due to the decreased utilization of the active material MnFe_2_O_4_@C. Specifically, the smaller fraction of the OH^−^ ions in electrolytes can intercalate into the structure of the MnFe_2_O_4_ at a higher scan rate than that of a low scan rate, which results in the decreased utilization of the active materials. In addition, GCD plots of the MnFe_2_O_4_@C electrode at various current densities are exhibited in Figure 4c. The shape of the GCD plot at any current density is triangular. The GCD plots are symmetric, hinting its good reversibility and agreement with the CV results. Similarly, the specific capacitances of the MnFe_2_O_4_@C electrode decrease with enhanced current density (Figure 4d). The maximum specific capacitance of the MnFe_2_O_4_@C electrode at 0.01 A·g^−1^ is 867 F·g^−1^. The specific capacitances at different current densities are 824, 746, 625, 508 and 476 F·g^−1^ from 0.1 to 5 A·g^−1^, about 57.8% capacitance retention. Long-term circulation is an essential criterion for evaluating supercapacitors. The cycling performance of the MnFe_2_O_4_@C electrode at 1 A·g^−1^ is shown in Figure 5. Apparently, only 6.1% specific capacitance loss is noticed after repeating charging-discharging for 10,000 cycles, illustrating its distinguished reversibility and stability.

**Figure 6:**
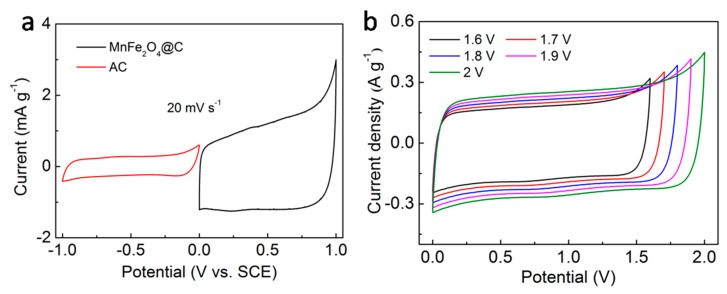
CV curves (**a**) of the MnFe_2_O_4_@C and AC electrodes measured in a three-electrode system, CV curves (**b**) at various working voltage of the MnFe_2_O_4_@C//AC device.

**Figure 7:**
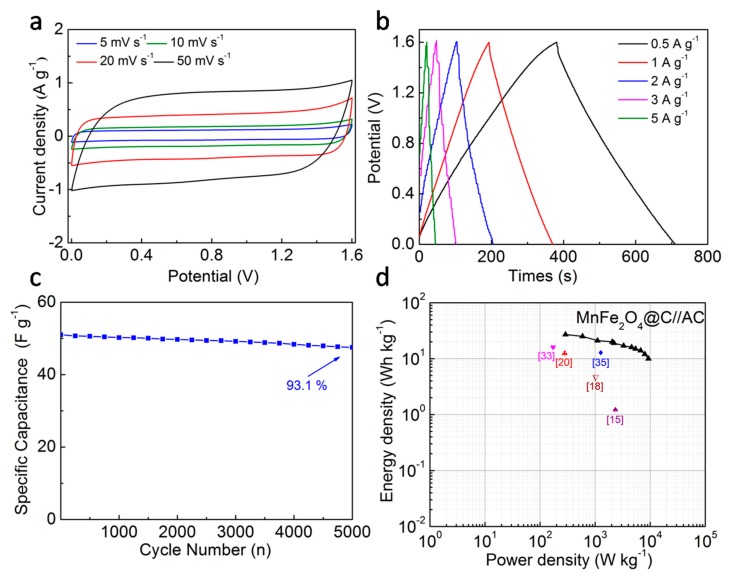
CV curves (**a**), GCD plots (**b**), long-term cycling performance at 1 A·g^−1^ (**c**), Ragone plots (**d**) of the MnFe_2_O_4_@C//AC device.

For practical application, the two-electrode system, MnFe_2_O_4_@C//AC device, has been assembled in our work. Figure 6a depicts the potential window of the MnFe_2_O_4_@C and AC electrodes at 20 mV·s^−1^ scan rate, respectively. So the theoretical working voltage window of the MnFe_2_O_4_@C//AC device is 2 V, according to the working voltage window of negative and positive electrodes. It is noted that the electrolyte of the MnFe_2_O_4_@C//AC device is 2 M KOH aqueous solution. To estimate the optimal operating potential window, the CV curves of the MnFe_2_O_4_@C//AC device operated under different potential windows at 10 mV·s^−1^ are shown in Figure 6b. It can be seen that when the voltage is higher than 1.7 V, the curve shows a pronounced polarization, corresponding to the oxygen evolution reaction in the electrolyte. So the operation window of 1.6 V is determined to be the suitable operating voltage. The CV curves of the MnFe_2_O_4_@C//AC device at various scan rates are depicted in Figure 7a. The shape of the CV curves has no obvious distortion and the corresponding current density increases with the enhanced scan rate, indicating its excellent rate performance. The GCD tests of the MnFe_2_O_4_@C//AC device at different current densities are performed (Figure 7b). The shapes of the GCD plots are almost the same, except for different discharging times. The cycling performance of the MnFe_2_O_4_@C//AC device at 1 A·g^−1^ is investigated and the results are depicted in Figure 7c. It can be seen that there is a slight decrease of the specific capacitance, about 6.9% capacitance decrement after 5000 cycles, confirming the outstanding long-term cycling performance of the MnFe_2_O_4_@C//AC device. Furthermore, the Ragone plot of the assembled MnFe_2_O_4_@C//AC deviceis obtained from calculating the results of GCD plots (Figure 7d). The MnFe_2_O_4_@C//AC device delivers a high energy density of 27 W·h·kg^−1^ at a power density of 290 W·kg^−1^, and remains at a 10 W·h·kg^−1^ energy density at a high power density of 9300 W·kg^−1^, which is higher than the previous reports [15,18,20,33,35]. The outstanding high-rate performance and cycling stability could be due to the following two aspects: (1) the unique porous, core-shell architecture and carbon coating’s mechanical and electrochemical stability, which contributes to fast electron and ion transportation and long-time charge-discharge measurements; (2) the uncontrolled space between core-shell nanoparticles or between the whole nanowires cantolerate the volume change from the fast and long-term electrochemical reactions.

## 4. Conclusions

We designed and fabricated novel MnFe_2_O_4_@C nanowires via a simple combination of the modified micro-emulsion technique and thermal treatment. The obtained MnFe_2_O_4_@C nanowires display about 200 nm diameter and 10 μm length. The in-situ carbon coating has been achieved, which enhances the whole electronic conductivity effectively and protects the MnFe_2_O_4_ nanoparticles from aggregation with charging-discharging procedures. And the experimental results demonstrate that the MnFe_2_O_4_@C electrode exhibits high specific capacitance, distinguished rate performance and stable cycling properties, which further verifies its candidacy as a supercapacitor electrode material.
